# MAFMamba: A Multi-Scale Adaptive Fusion Network for Semantic Segmentation of High-Resolution Remote Sensing Images

**DOI:** 10.3390/s26020531

**Published:** 2026-01-13

**Authors:** Boxu Li, Xiaobing Yang, Yingjie Fan

**Affiliations:** College of Information Engineering, China Jiliang University, Hangzhou 310018, China; p23030854078@cjlu.edu.cn (B.L.); p23030854072@cjlu.edu.cn (Y.F.)

**Keywords:** high-resolution remote sensing imagery, multi-scale fusion, semantic segmentation, Vision Mamba

## Abstract

With rapid advancements in sub-meter satellite and aerial imaging technologies, high-resolution remote sensing imagery has become a pivotal source for geospatial information acquisition. However, current semantic segmentation models encounter two primary challenges: (1) the inherent trade-off between capturing long-range global context and preserving precise local structural details—where excessive reliance on downsampled deep semantics often results in blurred boundaries and the loss of small objects and (2) the difficulty in modeling complex scenes with extreme scale variations, where objects of the same category exhibit drastically different morphological features. To address these issues, this paper introduces MAFMamba, a multi-scale adaptive fusion visual Mamba network tailored for high-resolution remote sensing images. To mitigate scale variation, we design a lightweight hybrid encoder incorporating an Adaptive Multi-scale Mamba Block (AMMB) in each stage. Driven by a Multi-scale Adaptive Fusion (MSAF) mechanism, the AMMB dynamically generates pixel-level weights to recalibrate cross-level features, establishing a robust multi-scale representation. Simultaneously, to strictly balance local details and global semantics, we introduce a Global–Local Feature Enhancement Mamba (GLMamba) in the decoder. This module synergistically integrates local fine-grained features extracted by convolutions with global long-range dependencies modeled by the Visual State Space (VSS) layer. Furthermore, we propose a Multi-Scale Cross-Attention Fusion (MSCAF) module to bridge the semantic gap between the encoder’s shallow details and the decoder’s high-level semantics via an efficient cross-attention mechanism. Extensive experiments on the ISPRS Potsdam and Vaihingen datasets demonstrate that MAFMamba surpasses state-of-the-art Convolutional Neural Network (CNN), Transformer, and Mamba-based methods in terms of mIoU and mF1 scores. Notably, it achieves superior accuracy while maintaining linear computational complexity and low memory usage, underscoring its efficiency in complex remote sensing scenarios.

## 1. Introduction

Remote sensing imagery serves as a critical data source for geospatial information, finding widespread application in various fields including environmental monitoring [1], urban planning [2], agricultural management [3], and disaster assessment [4]. Advancements in satellite and aerial imaging technologies have significantly lowered the barriers to accessing high-resolution imagery, offering unprecedented visual detail for fine-scale object recognition [5]. These high-resolution images provide not only sub-meter spatial resolution but also shorter revisit times, enabling clear depictions of object structures, contours, and contextual information. Consequently, they provide robust data support for both scientific research and industrial decision-making [4].

Semantic segmentation of high-resolution remote sensing imagery acts as a vital link between raw image data and practical applications. The objective is to assign precise land cover labels to each pixel, facilitating industrial land use planning, intelligent urban management, and automated surface information extraction. Traditional segmentation techniques, which rely heavily on low-level features such as spectra and textures, struggle to address issues related to intra-class heterogeneity and inter-class similarity in high-resolution images, often resulting in inadequate performance in complex environments [6]. As spatial resolution increases, these traditional methods encounter growing difficulties in accurately delineating boundaries and identifying small targets.

In recent years, deep learning has demonstrated impressive feature learning and abstraction capabilities in remote sensing image segmentation, particularly improving accuracy in sub-meter imagery tasks. Nevertheless, the intricate features and geometric details of high-resolution images remain difficult for medium- and low-resolution models to capture effectively. Moreover, a focus on high-level semantics can compromise edge and shape retention, negatively impacting overall classification accuracy [7,8]. Remote sensing scenes exhibit distinct multi-scale characteristics [9]; the same object categories often display significant variations in morphology and context across different scales, necessitating models that incorporate cross-scale joint modeling capabilities [10]. Current deep learning-based approaches for remote sensing image semantic segmentation generally fall into three categories: Convolutional Neural Networks (CNNs), Vision Transformers (ViTs), and emerging Mamba architectures grounded in State Space Models (SSMs).

### 1.1. Convolutional Neural Network Segmentation Methods

CNNs excel at detailed feature extraction due to their local receptive fields and weight-sharing mechanisms. Fully Convolutional Networks (FCN) [11] was the first to propose an end-to-end fully convolutional segmentation approach compatible with images of any size; however, its simple upsampling often leads to a loss of spatial details and blurred boundaries. U-Net [12] introduced a symmetric encoder–decoder architecture with skip connections for multi-level feature fusion, effectively compensating for down-sampling losses and becoming a mainstream structure for semantic segmentation. Subsequent works such as [13,14,15,16] further optimized CNN architectures; for example, the DeepLab series introduced Atrous Spatial Pyramid Pooling (ASPP) [17]; ABCNet [18] designed bilateral context attention modules to enhance edges; and MANet [19] improved road extraction efficiency from aerial imagery using lightweight multi-scale aggregation units.

In addition to architectural optimizations, novel learning paradigms such as masked supervised learning [20] have been proposed to force the network to capture more robust global contextual representations. Beyond remote sensing, advanced multi-scale feature fusion strategies have also emerged in general object and text detection tasks. For instance, AFPN [21] proposes an asymptotic feature pyramid network to facilitate direct interaction across non-adjacent levels. Similarly, CM-Net [22] utilizes concentric masks to capture distinct shape characteristics, while Zoom Text Detector [23] employs a zoom-in strategy to handle extreme scale variations. Although these methods demonstrate effective feature fusion capabilities, they are primarily tailored for instance-level detection or specific shape priors. In contrast, our strategy focuses on dense pixel-level semantic modeling, employing adaptive Coordinate Attention (CA) to dynamically recalibrate weights for the complex, unstructured scale variations inherent in remote sensing scenes. However, the fixed receptive field of CNNs limits their global context modeling capability, making it difficult to effectively distinguish intra-class heterogeneity and inter-class similarity in complex remote sensing scenes, thereby limiting segmentation performance.

### 1.2. Vision Transformer Segmentation Methods

To overcome the limitations of local constraints in convolutional feature extraction, Transformers were introduced into remote sensing image semantic segmentation tasks. Vision Transformers (ViT) [24] convert 2D image data into 1D sequences, utilizing self-attention mechanisms to model global dependencies between pixels, thereby effectively capturing global contextual information within images. Architectures relying solely on Transformers—such as Segmenter [25] and SegFormer [26]—have demonstrated superior performance over conventional CNNs by explicitly modeling global relationships. Hybrid structures combine the local feature extraction of CNNs with the global modeling advantages of Transformers. For example, FTransUNet [16] uses CNNs for shallow detail extraction and employs a three-stage fusion Transformer (FVit) for cross-modal global semantics, achieving complementary local–global feature integration. DC-Swin [27] and UNetFormer [28] utilize Swin Transformer [29] parallel branches and cross-scale attention to achieve local–global complementarity. However, the quadratic complexity of self-attention results in high inference memory usage for high-resolution images. Reformer [30] and Longformer [31] use sparse attention matrices, significantly reducing computational load and memory usage by selectively focusing on local or globally important information. Nevertheless, these methods inevitably lose some critical global contextual information, especially in high-resolution image processing, which weakens global feature extraction capabilities.

### 1.3. Mamba Segmentation Methods

The Mamba architecture [32] based on State Space Models (SSM), has recently emerged as a potential alternative to Transformers due to its linear computational complexity and proficiency in long-range dependency modeling, demonstrating distinct advantages in remote sensing image semantic segmentation tasks. Visual State Space Models (VSS) optimize scanning strategies to better suit visual tasks, significantly improving segmentation accuracy and efficiency. Various SSM-based Mamba models have been successfully deployed across diverse applications, including Vision Mamba [33], VMamba [34], PlainMamba [35], and MambaVision [36] for general vision tasks. Samba [37] pioneered the application of SSMs in remote sensing image semantic segmentation, substituting the multi-head self-attention mechanism of Vision Transformers to markedly enhance segmentation accuracy and inference efficiency without relying on pre-trained weights. RS3Mamba [38] introduced a dual-branch structure, merging CNN and VSS features through a pre-trained VSS auxiliary branch, surpassing existing methods but at the expense of increased computational costs due to its complex structure. CM-UNet [39] integrates a ResNet-18 encoder with a Mamba decoder, incorporating channel-wise spatial attention mechanisms to boost feature representation, though gating mechanisms may impact inference efficiency. Additionally, Pan-Mamba [40] and ChangeMamba [41] further validate the potential of VSS in multi-scale feature extraction from remote sensing images. Recent advancements, such as DefMamba [42] and ACMamba [43], refine local feature extraction through deformable windows and asymmetric consensus mechanisms, striving to balance accuracy and efficiency. However, current VSS-based approaches typically flatten 2D images into 1D sequences for global scanning, inherently disrupting spatial structural integrity. Consequently, while these methods excel at global modeling, they often neglect local feature expression and lack effective mechanisms for cross-scale interaction. This limitation results in two critical issues in complex remote sensing scenarios: (1) the loss of high-frequency local details (e.g., edges and small targets) due to the dominance of global context and (2) the inability to effectively represent objects with large-scale variations, leading to semantic inconsistencies.

To overcome the limitations associated with insufficient local detail modeling and inadequate multi-scale fusion, we propose a Multi-scale Feature Adaptive Fusion Mamba network (MAFMamba) for the semantic segmentation of high-resolution remote sensing imagery. MAFMamba employs a hybrid encoder–decoder architecture designed to synergistically integrate local, global, and multi-scale information. Our primary contributions are summarized as follows. Our primary contributions are as follows:

- **The MAFMamba Network:** We propose MAFMamba, a novel multi-scale adaptive fusion network tailored for high-resolution remote sensing imagery. Extensive experiments on the ISPRS Potsdam and Vaihingen benchmarks demonstrate that MAFMamba achieves state-of-the-art results in terms of both mean Intersection over Union (mIoU) and mean F1-score (mF1).
- **The Adaptive Multi-scale Mamba Block (AMMB):** To specifically mitigate the challenge of scale variation, we design the AMMB for the encoder. It incorporates a novel Multi-scale Adaptive Fusion (MSAF) mechanism that utilizes coordinate attention to dynamically recalibrate features across different levels. This ensures a robust representation of objects at varying scales, which is subsequently processed by VSSLayers to incorporate long-range dependencies.
- **The Global–Local Mamba Block (GLMamba):** To address the loss of local details, we introduce the GLMamba block for feature enhancement within the decoder. Adopting a dual-branch structure, it complements local fine-grained features (extracted via convolution) with global long-range dependencies (modeled via VSS), thereby ensuring precise boundary delineation and improved small object detection.
- **The Multi-Scale Cross-Attention Fusion Module (MSCAF):** To transcend the limitations of standard skip connections, we propose the MSCAF module. It supersedes traditional connections by employing an efficient cross-attention mechanism with linear complexity. MSCAF explicitly aligns and merges multi-scale features sourced from different encoder stages, effectively bridging the semantic gap and enhancing cross-scale consistency.

## 2. Methods

This section elaborates on the proposed MAFMamba framework. We first present the Overall Architecture (Section 2.1), followed by the core components: the Adaptive Multi-scale Mamba Block (AMMB, Section 2.2) in the encoder, the Global–Local Mamba Block (GLMamba, Section 2.3) and Adaptive Scale Fusion (ASF, Section 2.4) in the decoder, and the Multi-Scale Cross-Attention Fusion (MSCAF, Section 2.5) module that bridges these stages. Finally, we define the loss function and evaluation metrics.

### 2.1. Overall Architecture

**Figure 1:**
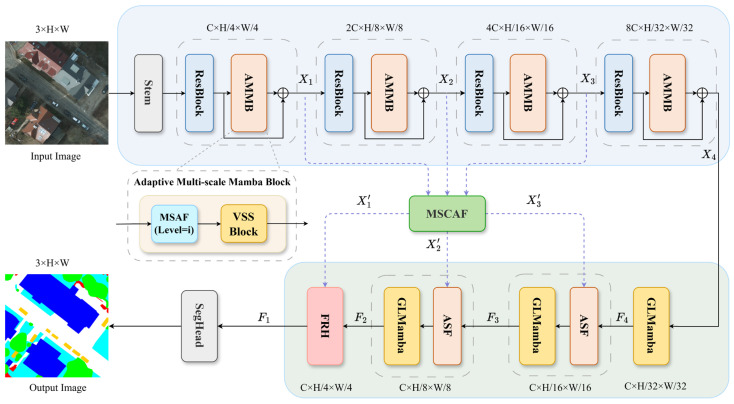
Overall architecture of MAFMamba.

Our proposed MAFMamba network adopts an encoder–decoder architecture, as illustrated in Figure 1. The overall framework constitutes a standard encoder–decoder network comprising three principal components: (1) a lightweight CNN-based encoder integrated with Adaptive Multi-scale Mamba Blocks (AMMB) for multi-scale feature extraction and global dependency modeling; (2) a Multi-Scale Cross-Attention Fusion (MSCAF) module designed to bridge the semantic gap between low-level encoder features and high-level decoder features; and (3) a decoder network incorporating Global–Local Mamba blocks (GLMamba) and an Adaptive Scale Fusion (ASF) module. Specifically, given an input remote sensing image, the encoder is built upon a ResNet-18 backbone. Initial features are extracted via a convolutional layer, followed by batch normalization, an activation function, and a max-pooling operation, resulting in a feature stride of 4. Subsequently, four residual blocks (Layer1–Layer4) generate feature maps with progressively decreasing spatial resolutions, denoted as $X_{i}\in R^{H_{i}\times W_{i}\times C_{i}}$ for $i\in \left(1,2,3,4\right)$, with dimensions defined as $H_{i}=H/2^{i+1}$, $W_{i}=W/2^{i+1}$, $C_{i}=2^{i-1}\times 64$. To facilitate the adaptive fusion of multi-scale features and enhance global modeling capabilities, an AMMB is inserted after each residual block. Leveraging the interaction between the Multi-scale Adaptive Fusion (MSAF) mechanism and the VSSLayer, the AMMB performs progressive multi-scale interaction modeling across the four encoder stages. This process yields a sequence of four comprehensively enhanced multi-scale features. The encoder outputs—specifically $X_{1},\;X_{2}$ and $X_{3}$, which carry rich multi-scale information—are subsequently fed into the MSCAF module. Within this module, a computationally efficient cross-attention mechanism facilitates bidirectional feature interaction, producing refined auxiliary features $X_{1}^{\prime },\;X_{2}^{\prime }$ and $X_{3}^{\prime }$. The decoder receives high-level features from the encoder’s top layer and sequentially processes them through the ASF module and the GLMamba block to progressively restore spatial resolution and refine feature representations. Within each GLMamba, the global branch employs a VSSLayer to capture long-range contextual information, while the local branch enhances sensitivity to edges, textures, and shadows through a dilated pyramid convolution structure. Both branches feed into a lightweight dual-branch adaptive fusion mechanism to achieve the complementary integration of global and local information. Additionally, the ASF module is employed during successive upsampling stages. It utilizes Softmax-generated weights to adaptively fuse high-level semantic information with low-level details, thereby ensuring effective resolution recovery while maintaining semantic consistency. Finally, the output features undergo spatial and channel optimization via the Feature Refinement Head (FRH), followed by the generation of pixel-level classification results through the Segmentation Head.

### 2.2. Adaptive Multi-Scale Mamba Block

Effective fusion of multi-scale semantic information and global context modeling are critical for the semantic segmentation of remote sensing images, particularly where objects of vastly different scales frequent co-occur. Conventional Convolutional Neural Networks (CNNs) are constrained by limited local receptive fields, which hinders cross-hierarchical multi-scale semantic alignment. Conversely, while Transformer-based architectures excel at capturing global relationships, they incur substantial computational overhead. Pure State Space Model (e.g., Mamba) variants, although efficient in modeling long-range dependencies, typically lack explicit mechanisms for globally integrating multi-scale information. Existing Feature Pyramid Network (FPN) variants, such as ASFF [44] and MFAF [45], facilitate cross-layer feature fusion. However, they commonly rely on simplistic operations—such as channel concatenation and 1×1 convolutions—to generate fixed fusion weights. This inability to adaptively recalibrate scale-wise contributions, based on local pixel context, often leads to semantic inconsistencies or blurred boundaries in regions exhibiting extreme scale variations, such as vehicles, narrow roads, and shadow edges.

To address these limitations, we introduce an Adaptive Multi-scale Mamba Block (AMMB), serving as a core enhancement unit appended after each encoder stage. The AMMB design centers on a synergistic, two-phase workflow involving a novel Multi-scale Adaptive Fusion (MSAF) module and a Vision State Space Layer (VSSLayer). This “fuse-then-model” paradigm follows a strategy of “local enrichment followed by global diffusion”: it first dynamically integrates features across scales via the MSAF to provide a spatially rich representation, and subsequently injects long-range dependencies through the VSSLayer. Although this sequential structure prioritizes bottom-up refinement, we incorporate residual connections to prevent information bottlenecks and preserve original feature cues. Consequently, at each encoder stage, the AMMB achieves a cohesive integration of local fine-grained detail preservation, adaptive multi-scale fusion, and global dependency modeling. This approach effectively mitigates the issues of semantic inconsistency and boundary ambiguity prevalent in complex remote sensing scenarios.

#### 2.2.1. Multi-Scale Adaptive Fusion Module

The MSAF serves as the initial stage within the AMMB. Its principal innovation is the adoption of a Coordinate Attention mechanism to facilitate pixel-wise dynamic weight generation. This approach supersedes conventional fusion strategies that rely on fixed or channel-wise weighting, enabling genuinely adaptive fusion across both spatial and scale dimensions. While inspired by prior works such as ASFF [44] and the MFAF module in AfaMamba [45], our key optimization lies in the weight generation mechanism. Specifically, we transcend simple convolutional operations or channel attention methods by incorporating a lightweight coordinate attention block. This design enables the concurrent capture of inter-channel dependencies and spatial positional cues, yielding precise, spatially aware fusion weights.

**Figure 2:**
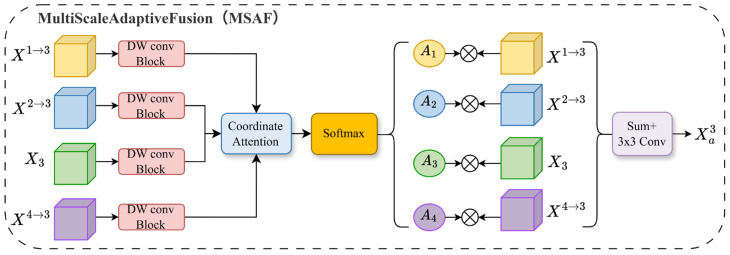
Architecture of the Multi-scale Adaptive Fusion (MSAF) module, illustrated using features from the third encoding stage.

Specifically, at each encoding stage, the MSAF module receives features from all hierarchical levels, denoted as $\left\{X_{1},X_{2},X_{3},X_{4}\right\}$. Leveraging a coordinate attention mechanism for cross-scale adaptive fusion, it produces a semantically enhanced feature map $X_{a}^{i}$ that maintains the same spatial resolution as the current level $X_{i}$. To facilitate understanding, we detail the process using Stage 3 as a representative example, as shown in Figure 2. The procedure is as follows:

**Feature Scale Unification:** To facilitate effective cross-scale information fusion, the first step involves resizing all input feature maps $X_{j}\in R^{C_{j}\times H_{j}\times W_{j}}$ uniform spatial resolution, matching the target scale of the current stage (i.e., the dimensions of $X_{3}$ where H/16×W/16). We employ an adaptive resizing strategy tailored to the resolution of each input feature map. For feature maps with higher resolution than the target (e.g., $X_{1}$, $X_{2}$), channel numbers are first adjusted via a 1×1 convolution, followed by spatial downsampling using bilinear interpolation with a scale factor s. Conversely, for feature maps of lower resolution (e.g., $X_{4}$), a strided convolutional layer is applied for initial feature transformation, followed by bilinear interpolation for upsampling. The feature map $X_{3}$, already at the target resolution, undergoes channel adjustment via a 1×1 convolution. This procedure ensures the precise alignment of all feature maps in both spatial and channel dimensions, establishing a consistent foundation for subsequent adaptive weighted fusion. The overall operation can be formulated as follows:

(1) $$X^{1\to 3}={Interpolate}_{s=0.25}({Conv}_{1\times 1}(X_{1}))$$

(2) $$X^{2\to 3}={Interpolate}_{s=0.5}({Conv}_{1\times 1}(X_{2}))$$

(3) $$X_{3}={Conv}_{1\times 1}(X_{3})$$

(4) $$X^{4\to 3}={Interpolate}_{size(X_{2})}({Conv}_{3\times 3,s=2}(X_{4}))$$

Following this step, we obtain four feature maps of consistent dimensions: $X^{1\to 3}$, $X^{2\to 3}$, $X_{3}$, $X^{4\to 3}$.

**Enhanced Weight Feature Extraction:** To produce spatially aware fusion weights, the MSAF module incorporates a lightweight enhancement branch for each of the four spatially aligned feature maps $\left\{X^{1\to 3},\;X^{2\to 3},X_{3},\;X^{4\to 3}\right\}$. This branch sequentially applies a 3×3 depthwise separable convolution, batch normalization, and a ReLU activation function to capture localized spatial context. Subsequently, a 1×1 convolutional layer projects the extracted features into a lower-dimensional space (e.g., 16 channels), yielding a compact representation termed the weight feature map $W_{k}^{\prime }$ (where k∈{1,2,3,4}):

(5) $$W_{k\;}^{\prime }={Conv}_{1\times 1}\left(ReLU\left({DWConv}_{3\times 3}(X^{k\to 3})\right)\right)$$

This design effectively enhances the spatial representational capacity of weight prediction with negligible additional computational overhead.

**Adaptive Weight Generation:** In contrast to conventional channel attention mechanisms, the MSAF module employs coordinate attention to produce spatially adaptive fusion weights. Specifically, the set of four weight feature maps $\left\{W_{1}^{\prime },W_{2}^{\prime },W_{3}^{\prime },W_{4}^{\prime }\right\}$ is first concatenated. The resulting tensor is then fed into a coordinate attention block, which independently captures long-range spatial dependencies along the horizontal and vertical axes. This process yields four pixel-wise fusion weight maps, formulated as follows:

(6) $$W_{cat}=Concat(W_{1}^{\prime },W_{2}^{\prime },W_{3}^{\prime },W_{4}^{\prime })$$

(7) $$\left[A_{1},A_{2},A_{3},A_{4}]=Softmax(CA(W_{cat})\right)$$

**Weighted Fusion and Feature Enhancement:** The generated spatial weights are applied to the aligned multi-scale features to compute their pixel-wise weighted sum. This aggregated representation is then refined by a 3×3 convolutional layer, ultimately producing the stage-specific, adaptively fused feature $X_{a}^{3}$.

(8) $$X_{a}^{3}={Conv}_{3\times 3}\left(\sum_{k=1}^{4}\;A_{k}⊙X^{k\to 3}\right)$$

where ⊙ denotes the element-wise multiplication, enabling the spatial weights $A_{k}$ to dynamically recalibrate the feature maps $X^{k\to 3}$ at the pixel level before aggregation.

Although the output $X_{a}^{i}$ of the MSAF module fuses multi-scale information, its receptive field is essentially limited by the convolutional operation. To capture global long-range dependencies, we feed $X_{a}^{i}$ into the corresponding Vision State Space Layer.

#### 2.2.2. Visual State Space Layer

While the MSAF-generated feature $X_{a}^{i}$ incorporates multi-scale cues, its effective receptive field remains constrained by the locality of convolutional operations. To compensate for this limitation and enable comprehensive global dependency modeling, $X_{a}^{i}$ is processed by a subsequent Vision State Space Layer (VSSLayer). As established in prior work [30], the VSSLayer—specifically its core 2D Selective Scan module (SS2D)—constitutes a pivotal mechanism for establishing long-range contextual interactions. Within this layer, the input feature map is first unfolded into a sequential representation. At the core of the VSSLayer lies the Linear State Space Model (SSM), which can be represented by a linear ordinary differential equation (ODE) to model continuous system dynamics:

(9) $$h^{\prime }(t)=Ah(t)+Bx(t),\;y(t)=Ch(t)+Dx(t)$$

where A represents the evolution parameter, B and C are projection parameters, and D is the parameter for the skip connection. $x\left(t\right)$ denotes the input sequence, $h\left(t\right)$ represents the hidden state, and $h^{\prime }\left(t\right)$ indicates the derivative of the hidden state. However, as the VSSM processes discrete 1-D image sequences, the continuous parameters A and B must be discretized using the Zero-Order Hold (ZOH) method. Specifically, a timescale parameter Δ is introduced to transform the continuous parameters into their discrete counterparts $\bar{A}$ and $\bar{B}$:

(10) $$\bar{A}=\exp\left(\Delta A\right),\;\bar{B}={\left(\Delta A\right)}^{-1}\left(\exp\left(\Delta A\right)-I\right)\Delta B$$

After discretization, Equation (9) can be rewritten in the recursive RNN form suited for efficient inference:

(11) $$h_{t}=\bar{A}h_{t-1}+\bar{B}x_{t},\;y_{t}=Ch_{t}+Dx_{t}$$

The core component of the VSSLayer is the 2D Selective Scan (SS2D) module. The SS2D module processes the input 2D feature map F by performing selective scanning along four principal directions (upward, downward, leftward, and rightward), thereby modeling long-range spatial dependencies across varying propagation paths. The detailed workflow of the SS2D module is described below:

First, the input feature F undergoes Patch Expansion to widen its receptive field, followed by layer normalization and channel projection through a linear embedding layer:

(12) $$\begin{array}{r} F^{\prime }=LinearEmbed\left(LayerNorm\left(PatchExp\left(F\right)\right)\right) \end{array}$$

A 3×3 depthwise separable convolution is then applied to enhance local feature responses, followed by SiLU activation to obtain an enriched representation:

(13) $$\begin{array}{r} F^{\prime \prime }=SiLU\left(DWConv\left(F\prime \right)\right) \end{array}$$

Next, directional state sequences are extracted by scanning F″ along the four predefined directions:

(14) $$\begin{array}{r} F_{v}=ScanExp\left(F\prime \prime ,v\right),\;v\in \{1,2,3,4\} \end{array}$$

Each directional sequence is processed by the S6 (Selective Scan State Space) model to capture long-range dependencies, after which the resulting representations are merged:

(15) $$\tilde{F}=ScanMerge(F_{1},F_{2},F_{3},F_{4})$$

In parallel, a bypass branch processes $F^{\prime }$ through another linear embedding and SiLU activation to produce an auxiliary feature map. The main output $\tilde{F}$ is normalized and then fused with the bypass output via element-wise multiplication, after which a residual connection from the original input F is added:

(16) $$F_{out}=Linear(LN(\tilde{F})⊙F_{bypass})+F$$

where ⨀ denotes the element-wise Hadamard product. The adaptively fused feature $X_{a}^{i}$ is subsequently passed into the corresponding VSSLayer. This layer effectively integrates global contextual information into multi-scale features, thereby strengthening representation of large objects and complex structural patterns:

(17) $$X_{vss}^{i}={VSSLayer}_{i}(X_{a}^{i})$$

To retain the high-frequency spatial details from the original encoder features and promote training stability, a residual connection is employed to combine the VSSLayer output $X_{vss}^{i}$ with the encoder feature $X_{i}$ from the same stage:

(18) $$X_{out}^{i}=X_{vss}^{i}+X_{i}$$

The $X_{out}^{i}$ denotes the final output of the AMMB module at stage i. It integrates multi-scale semantic information aggregated across different levels by the MSAF, is equipped with global context awareness via the VSSLayer, and retains the original fine-grained features.

Within the MAFMamba encoder, the VSSLayer and the MSAF module constitute the AMMB feature enhancement unit. This unit provides the subsequent decoder with a multi-scale feature sequence that is semantically coherent, rich in detail, and globally aware. In the decoder, the global branch leverages the VSSLayer to model long-range dependencies, thereby capturing broad-context information within the remote sensing imagery. The VSSLayer is built upon the visual state space mechanism of the Mamba model. Its primary objective is to capture global context and model long-range dependencies, addressing the constraint of the limited receptive field inherent in traditional convolutional operations. This capability contributes to enhanced segmentation accuracy of the MAFMamba network.

### 2.3. Global–Local Feature Enhancement Mamba

**Figure 3:**
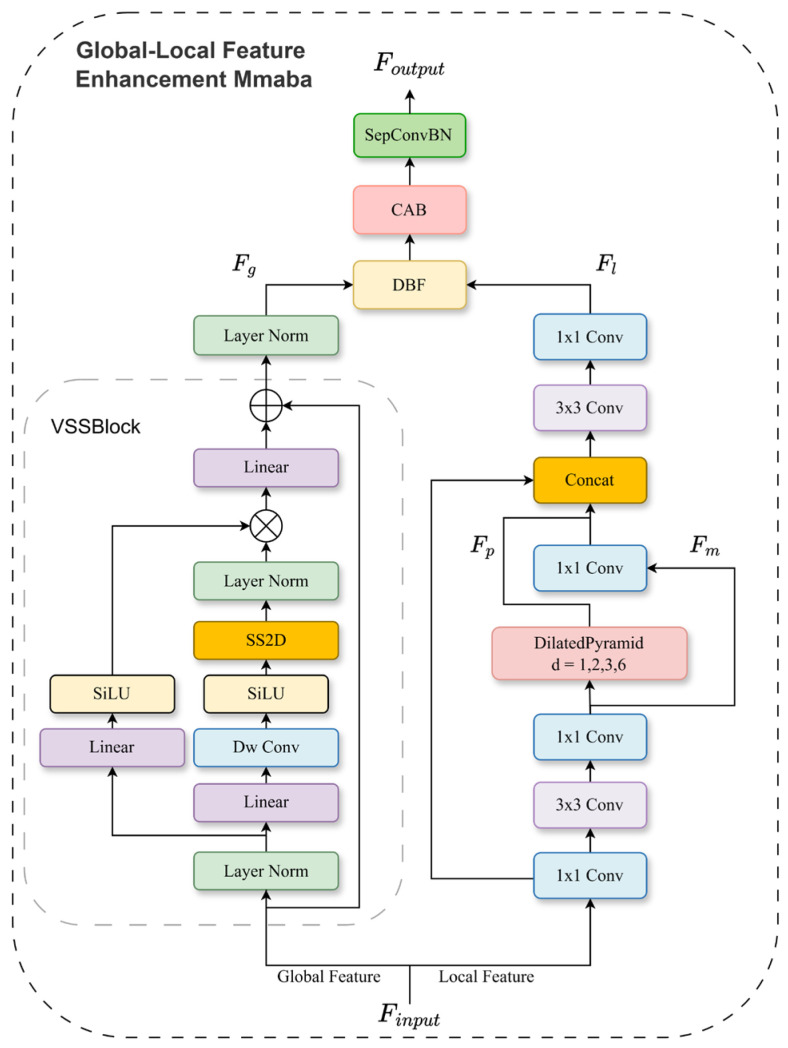
Structure of the Global–Local Feature Enhancement Mamba (GLMamba) module.

To fully exploit both local structural details and global contextual information in high-resolution remote sensing imagery, we design a Global–Local Feature Enhancement Mamba (GLMamba) module. It is tailored for the decoder stage to synergistically bolster local texture preservation and long-range dependency modeling. Unlike traditional approaches that simply concatenate local and global pathways, our GLMamba employs a dual-branch parallel architecture. Specifically, one branch extracts features via a local multi-scale convolutional path, while the other captures dependencies through a global state space modeling path. These complementary features are then dynamically integrated via an adaptive fusion mechanism. This design empowers the model to better perceive complex land-cover structures, fine edge details, and long-range semantic relationships. The overall structure of the GLMamba module is illustrated in Figure 3.

**Local Branch:** As illustrated in the right branch of Figure 3, the local branch is designed to capture multi-scale contextual features. To achieve this efficiently, it first employs a bottleneck structure. This bottleneck expands the input features into a higher-dimensional space with minimal computational overhead, followed by feature encoding. This step yields a richer joint representation of local semantics without increasing channel complexity for subsequent operations. Subsequently, a Dilated Pyramid Module (DPM) is introduced. This module utilizes parallel 3×3 dilated convolutions with different dilation rates (1, 2, 3, 6) to capture contextual information at multiple scales. All branches maintain the original spatial resolution. Features from each branch undergo batch normalization and ReLU activation for calibration. The resulting multi-scale features are then concatenated along the channel dimension and fused into a unified representation via a 1×1 convolution. This design significantly enhances the model’s sensitivity to structures of varying scales, while preserving spatial information that is often lost in pooling operations. Given an input feature $F_{in}\;\text{∈}R^{H\text{×}W\text{×}C}$, the overall modeling process of the local branch can be formulated as follows:

(19) $$F_{m}=\delta_{1\times 1}\left(\delta_{3\times 3}\left(\delta_{1\times 1}(F_{in})\right)\right)$$

(20) $$F_{p}=DPM(F_{m})=\psi_{fuse}\left(\delta_{3\times 3}^{\left(r\right)}(F_{m})|r\in \{1,2,3,6\}\right)$$

(21) $$F_{l}=\delta_{1\times 1}\left(\delta_{3\times 3}\left(Concat\left[F_{p},\delta_{1\times 1}(F_{m}),\delta_{1\times 1}(F_{in})\right]\right)\right)$$

where $F_{m}$ denotes the intermediate feature projected into a high-dimensional space for refined encoding, $\delta_{k\times k}\left(\cdot \right)$ denotes a composite operation consisting of a k×k convolution, followed by Batch Normalization and a ReLU activation function. DPM denotes the Dilated Convolution Pyramid Module, where $\delta_{3\times 3}^{\left(r\right)}$ indicates a 3×3 dilated convolution with a dilation rate r. $\psi_{fuse}$ is a 1×1 convolution that integrates the multi-branch outputs. The resultant local branch output $F_{l}$, thus integrates rich fine-grained details with effective multi-scale contextual awareness.

Global Branch: This branch is designed to capture long-range dependencies and global contextual information from the input feature maps, as illustrated on the left side of Figure 3. Its core component is the VSSLayer (detailed in Section 2.2.2), which effectively processes global information while maintaining computational efficiency. The input features are first reshaped to suit the VSSLayer’s operation. After global modeling, they are projected back to the original spatial dimensions, thereby enhancing the feature representation without loss of spatial resolution. The output of the global branch is denoted as follows:

(22) $$F_{g}=LayerNorm(VSSLayer(F_{in}))$$

**Feature Fusion Mechanism:** To effectively integrate the local branch output $F_{l}$ and the global branch output $F_{g}$, we propose an adaptive dual-branch fusion mechanism augmented by a channel-spatial attention module for refinement. The fusion process begins by concatenating $F_{l}$ and $F_{g}$ along the channel dimension. A global average pooling layer then compresses this concatenated feature into a 1×1×2C descriptor v. This descriptor passes through two consecutive 1×1 convolutional layers (with a ReLU activation in between) to reduce its dimensionality to 2, followed by a Softmax function to generate a pair of spatially shared adaptive weights $\left[\alpha ,\beta \right]$. These weights are applied to perform an adaptive fusion:

(23) $$v=AvgPool(Concat(F_{l},F_{g}))$$

(24) $$[\alpha ,\beta ]=Softmax\left({Conv}_{1\times 1}\left(ReLU\left({Conv}_{1\times 1}\left(v\right)\right)\right)\right)$$

(25) $$F_{fused}=\alpha \cdot F_{l}+\beta \cdot F_{g}$$

where $F_{fused}$ denotes the preliminary fused feature map obtained by the adaptive weighted summation of local and global branches. This adaptive, sample-wise and channel-wise weighting allows the network to dynamically balance the contributions of fine-grained details and global semantics. It effectively mitigates the discrepancies caused by distributional or scale mismatches between the two feature types—a common issue with simple concatenation or fixed-weight fusion—thereby achieving more stable integration and improved semantic coherence.

To further suppress noise and accentuate informative regions, $F_{fused}$ is refined by a Channel-Spatial Attention Block (CAB), yielding the final output:

(26) $$F_{output}=SeparableConvBN\left(CAB(F_{fused})\right)$$

where CAB sequentially applies channel attention and spatial attention to adaptively recalibrate feature importance across their respective dimensions. The subsequent depthwise separable convolution (with batch normalization) efficiently aggregates multi-scale contextual information while maintaining a low parameter count.

### 2.4. Adaptive Scale Fusion

**Figure 4:**
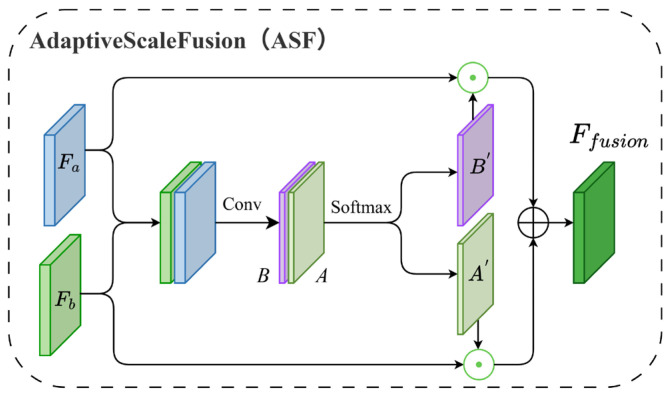
Structure of the Adaptive Scale Fusion (ASF) module.

To effectively integrate multi-level features during the decoding phase—where high-resolution features possess rich spatial details but weak semantics, while low-resolution features contain strong semantics but lack spatial definition—we propose an Adaptive Scale Fusion (ASF) module. As illustrated in Figure 4, the ASF balances detailed and semantic information by fusing multi-scale features via adaptive weighting.

The ASF module accepts two primary inputs: (1) the three-stage feature maps (denoted as $X_{1}^{\prime }$, $X_{2}^{\prime }$, $X_{3}^{\prime }$) generated by the Multi-Scale Cross-Attention Fusion (MSCAF) module, and (2) the feature map $F_{i}$ from the preceding decoder stage, with i∈{1,2,3}. These features are first aligned in terms of channel dimensions and spatial resolution. Subsequently, they are concatenated and processed by the ASF module. Internally, the ASF employs convolutional layers followed by a Softmax activation to generate two spatial attention maps, $A^{\prime }$ and $B^{\prime }$. These maps are utilized to adaptively weight the features for fusion. Specifically, a weighted summation is performed based on the learned attention weights:

(27) $$F_{fusion}=A^{\prime }⊙F_{A}+B^{\prime }⊙F_{B}$$

where ⨀ denotes the element-wise Hadamard product. $A^{\prime }$ and $B^{\prime }$ represent the learned spatial attention weights, while $F_{A}$ and $F_{B}$ denote the low-resolution and high-resolution features, respectively, which have undergone dimensionality reduction and upsampling. Finally, the fused features undergo refinement via convolution, Batch Normalization, and ReLU activation. This step eliminates redundancy introduced during fusion and enhances nonlinear expressive power, yielding the final fused feature map $F_{fusion}$.

### 2.5. Multi-Scale Cross-Attention Fusion Module

In semantic segmentation tasks, the encoder–decoder architecture has established itself as the dominant paradigm. This architecture progressively downsamples input data via the encoder to extract high-level semantic features, and subsequently upsamples via the decoder to restore spatial resolution. While traditional skip connections preserve the spatial details of high-resolution features, they frequently overlook the significant semantic gap existing between shallow encoder features (e.g., $X_{1}$) and deep features (e.g., $X_{2}$). Shallow features are characterized by rich spatial detail but weak semantics, whereas deep features possess abundant semantic information yet suffer from low spatial resolution. Direct fusion often precipitates semantic inconsistencies and information redundancy, thereby introducing noise and irrelevant context. Consequently, this results in issues such as blurred object boundaries and the failure to detect small objects, ultimately limiting segmentation accuracy.

To mitigate these issues, we propose the Multi-Scale Cross-Attention Fusion (MSCAF) module. The core objective of this module is to supersede traditional skip connections with an efficient cross-attention mechanism, thereby facilitating explicit interaction and information alignment between features at different scales. This design not only bridges the semantic gap between hierarchical features but also enables features at each scale to pre-incorporate contextual information from other scales prior to decoding, thus enhancing semantic consistency and feature complementarity.

The MSCAF module takes as input features from three encoder stages: $X_{i}\in R^{C_{i}\times H_{i}\times W_{i}}$, where i∈{1,2,3}. It facilitates bidirectional information aggregation between these features through pairwise efficient cross-attention. The detailed workflow is formulated as follows:

**Query, Key, and Value Projection:** The query feature $X_{i}$ (target scale) and context feature $X_{j}$ (source scale, where j≠i) are independently projected onto the query, key, and value subspaces via separate 1×1 convolutions:

(28) $$Q_{i}=W_{q}X_{i};{\;K}_{j}=W_{k}X_{j};{\;V}_{j}=W_{v}X_{j}$$

In this equation, $W_{q},W_{k},W_{v}$ are the learnable weight matrices of the 1×1 convolutions. $Q_{i}\in R^{C_{k}\times H_{i}\times W_{i}}$ represents the query feature map containing the target spatial structure, while $K_{j}\in R^{C_{k}\times H_{j}\times W_{j}}$ and $V_{j}\in R^{C_{v}\times H_{j}\times W_{j}}$ represent t the key and value feature maps providing contextual information from the source scale. To reduce computational complexity, we apply a dimension reduction rate of r=4 (i.e., $C_{v}=C_{i}/4$).

**Efficient Cross-Attention Computation:** Traditional dot-product attention necessitates the construction of a correlation matrix with dimensions $O\left(N_{i}N_{j}\right)$, where computational overhead increases significantly with resolution, rendering it unsuitable for remote sensing scenarios. Adopting the efficient attention mechanism utilized in [46], MSCAF reduces the quadratic complexity of standard cross-attention to linear complexity. Specifically, we first apply a column-wise Softmax along the channel dimension to the key matrix to derive a global context descriptor, followed by a row-wise Softmax along the spatial dimension of the query matrix for information retrieval:

(29) $$X_{i\leftarrow j}=\rho_{row}(Q_{i})\cdot (\rho_{col}(K_{j}{)}^{⊤}V_{j})$$

where ${\;\rho }_{row}$ and ${\;\rho }_{col\;}$ represent the application of the Softmax function independently to each row and column of the matrix, respectively, forming the spatial query weights and context-scale semantic summaries. This formulation reduces the computational complexity from $O\left(N_{i}N_{j}\right)$ to $O\left(N_{i}+N_{j}+C^{2}\right)$, thereby ensuring the feasibility of high-resolution remote sensing feature fusion.

**Bidirectional Aggregation and Enhancement:** To achieve comprehensive cross-scale information exchange, MSCAF performs bidirectional efficient cross-attention computations for all feature pairs. Upon completion, each original feature is fused with its aggregated contextual information from other scales. Specifically, for each scale’s feature $X_{i\;}$, we concatenate it with all attention results $\left\{X_{i\leftarrow j}\right\}$ obtained using it as the query source. This concatenated feature then passes through a 1×1 convolutional layer for channel fusion and dimensionality reduction, generating the final enhanced feature $X_{i}^{\prime }$:

(30) $$X_{i}^{\prime }={Conv}_{1\times 1}\left(Concat\left[X_{i},\{X_{i\leftarrow j}{\}}_{j\neq i}\right]\right)$$

**Figure 5:**
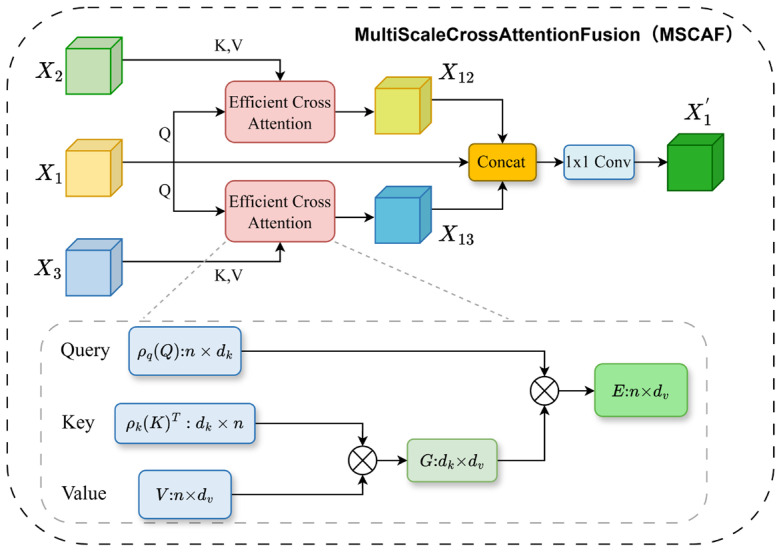
Structure of the Multi-Scale Cross-Attention Fusion (MSCAF) module (illustrating the enhancement process of feature $X_{1}$).

The module ultimately outputs three enhanced feature maps $\left\{X_{1}^{\prime },X_{2}^{\prime },X_{3}^{\prime }\right\}$, For instance, as illustrated in Figure 5 (taking i=1), the enhanced feature $X_{1}^{\prime }$ is generated by aggregating complementary semantic contexts from $X_{2}$ and $X_{3}$. These features preserve their intrinsic strengths while absorbing complementary information from other scales, providing the decoder with more robust and informative inputs. This significantly boosts segmentation accuracy in complex remote sensing scenes.

### 2.6. Loss Function

To enhance semantic segmentation performance on remote sensing imagery, we employ a composite loss function that integrates Soft Cross-Entropy (SCE) loss with Dice loss. This design jointly optimizes both pixel-wise classification accuracy and region-wise overlap consistency, thereby addressing segmentation challenges at varying granularities. The combined loss is formulated as

(31) $$L_{joint}=\lambda_{1}L_{sce}+\lambda_{2}L_{dice}$$

where $\lambda_{1}$ and $\lambda_{2}$ are weighting coefficients that balance the two loss terms. Based on sensitivity analysis, we empirically set $\lambda_{1}$ = $\lambda_{2}$ = 1.0, as this configuration yields optimal performance by effectively weighing pixel-level classification against region-level consistency. the SCE loss ($L_{sce}$) is defined as

(32) $$L_{sce}=-\frac{1}{N}\sum_{n=1}^{N}\sum_{k=1}^{K}y_{k}^{\left(n\right)}log{\hat{y}}_{k}^{\left(n\right)}$$

The Dice loss ($L_{dice}$) is expressed as

(33) $$L_{dice}=1-\frac{2}{N}\sum_{n=1}^{N}\sum_{k=1}^{K}\frac{y_{k}^{\left(n\right)}{\hat{y}}_{k}^{\left(n\right)}}{{\hat{y}}_{k}^{\left(n\right)}+y_{k}^{\left(n\right)}}$$

where N and K denote the number of pixels and categories, respectively, $y_{k}^{\left(n\right)}$ represents the binary ground truth label, while ${\hat{y}}_{k}^{\left(n\right)}$ indicates the predicted confidence that pixel n belongs to category k. By jointly optimizing the cross-entropy and Dice components, the model learns category discrimination from a probability distribution perspective while simultaneously enhancing boundary segmentation accuracy through regional overlap optimization.

### 2.7. Evaluation Metrics

To comprehensively evaluate the segmentation performance of the proposed model, we employ three standard metrics: mean Intersection over Union (mIoU), Overall Accuracy (OA), and mean F1-Score (mF1). Specifically, mIoU quantifies the average spatial overlap between predicted and ground truth regions across all categories. OA measures global pixel-wise classification accuracy. mF1, representing the harmonic mean of per-class precision and recall, offers a robust assessment, particularly in the presence of class imbalance. The mathematical formulations for these metrics are defined as follows:

(34) $$mIoU=\frac{1}{K}\sum_{k=1}^{K}\frac{{TP}_{k}}{{TP}_{k}+{FP}_{k}+{FN}_{k}}$$

(35) $$OA=\frac{{\sum_{k=1}^{K}TP}_{k}}{\sum_{k=1}^{K}({TP}_{k}+{FP}_{k}+{TN}_{k}+{FN}_{k})}$$

(36) $$mF1=\frac{1}{K}\sum_{k=1}^{K}\;\frac{2P^{\left(k\right)}R^{\left(k\right)}}{P^{\left(k\right)}+R^{\left(k\right)}}$$

(37) $$P^{\left(k\right)}=\frac{{TP}_{k}}{{TP}_{k}+{FP}_{k}},\;R^{\left(k\right)}=\frac{{TP}_{k}}{{TP}_{k}+{FN}_{k}}$$

where K denotes the number of classes in the dataset, ${TP}_{k}$, ${FP}_{k}$, ${TN}_{k}$, and ${FN}_{k}$ represent the number of true positive, false positive, true negative, and false negative pixels for class k, respectively.

## 3. Experiment

In this section, we first detail the Experimental Setup (Section 3.1) and Datasets (Section 3.2). Subsequently, we present comprehensive Performance Comparisons (Section 3.3) against state-of-the-art methods on the ISPRS Potsdam, Vaihingen, and LoveDA datasets. To validate the effectiveness of our core components, we conduct Ablation Studies (Section 3.4). Finally, we provide a Complexity Analysis (Section 3.5) to assess the model’s computational efficiency.

### 3.1. Experimental Setup

All experiments were conducted on NVIDIA Tesla V100 GPUs (32 GB VRAM, Nvidia, Santa Clara, CA, USA) using the PyTorch 2.1.0 framework with Python 3.10 and CUDA 12.1. We adopted a consistent training protocol across all models. The network was optimized using the AdamW optimizer with an initial learning rate of $6\times 10^{-4}$ and a weight decay of 0.01. The parameters of the pre-trained backbone were fine-tuned at a reduced learning rate of $6\times 10^{-5}$. The learning rate was scheduled via a cosine annealing restart strategy. Training was performed for 80 epochs with a batch size of 4. To enhance model robustness, we applied extensive data augmentation during training. This included random horizontal/vertical flips, random rotations by multiples of $90^{{}^{\circ}}$, and random resizing with scaling factors sampled from {0.5, 0.75, 1.0, 1.25, 1.5}. Furthermore, Smart Crop and mosaic augmentation were incorporated with a probability of 0.25. All training images were resized to a fixed resolution of 1024 × 1024. During inference, we employed random flipping and multi-scale evaluation to ensure fair performance assessment.

To guarantee a rigorous comparison, all baseline methods—encompassing CNN-based, Transformer-based, and Mamba-based models—were retrained using their official open-source implementations. We strictly unified the experimental protocols: all methods were trained on the same hardware with identical input resolutions (1024×1024), data augmentation strategies, and optimizer settings.

### 3.2. Dataset

To evaluate the performance of the proposed MAFMamba, experiments were conducted using three publicly available remote sensing semantic segmentation datasets: ISPRS Vaihingen, ISPRS Potsdam, and LoveDA.

**ISPRS Potsdam Dataset:** The Potsdam dataset comprises 38 True Orthophotos (TOP), each measuring 6000 ×6000 pixels with a Ground Sampling Distance (GSD) of 5 cm. The dataset encompasses six object categories: Impervious Surfaces, Building, Low Vegetation, Tree, Vehicle, and Clutter (background). It provides four spectral bands (Red, Green, Blue, and Near-Infrared) alongside a Digital Surface Model (DSM). In our experiments, only the RGB bands were utilized. We strictly followed the official benchmark partition: 24 images were used for training, while the remaining 14 images (IDs: 2_13, 2_14, 3_13, 3_14, 4_13, 4_14, 4_15, 5_13, 5_14, 5_15, 6_13, 6_14, 6_15, 7_13) were reserved exclusively for testing. All images were cropped into 1024×1024 pixel patches.

**ISPRS Vaihingen Dataset:** The Vaihingen dataset consists of 33 high-resolution image tiles, averaging 2494 ×2064 pixels with a GSD of 9 cm. It includes five foreground categories (Impervious Surfaces, Building, Low Vegetation, Tree, Vehicle) and one background category (Clutter). The dataset provides three multispectral bands (Near-Infrared, Red, Green) as well as DSM and normalized DSM (nDSM) data. In our study, only the Near-Infrared (IR), Red, and Green bands were utilized. To adhere to the official protocol and prevent data leakage, we divided the dataset into mutually exclusive subsets: 17 tiles (IDs: 2, 4, 6, 8, 10, 12, 14, 16, 20, 22, 24, 27, 29, 31, 33, 35, and 38) were reserved for the Test Set. The remaining 16 tiles formed the Training Set. For hyperparameter optimization, we randomly partitioned 20% of the training patches to constitute a Validation Set, ensuring no spatial overlap between the training/validation samples and the Test Set. All tiles were cropped into 1024×1024 pixel patches.

**LoveDA Dataset:** The LoveDA dataset contains 5987 high-resolution optical remote sensing images, each measuring 1024 ×1024 pixels with a GSD of 0.3 m. Unlike the urban-centric ISPRS datasets, LoveDA encompasses both urban and rural landscapes collected from three different cities (Nanjing, Changzhou, and Wuhan). This diversity introduces significant challenges related to multi-scale objects, complex background clutter, and inconsistent class distributions. The dataset defines seven land-cover categories: Background, Building, Road, Water, Barren, Forest, and Agriculture. Only the RGB images were utilized in our experiments. We strictly adhered to the official data partition, using 2522 images for training, 1669 for validation, and 1796 for testing. All images were used as model inputs at their original resolution of 1024 ×1024 pixels.

### 3.3. Performance Comparison

The quantitative comparisons of MAFMamba against state-of-the-art models on the ISPRS Vaihingen, Potsdam, and LoveDA datasets are summarized in Table 1, Table 2, and Table 3, respectively. The comparative analysis encompasses three distinct paradigms: (1) CNN-based models, including ESDINet [47], BANet [48], and MANet [19]; (2) CNN–Transformer hybrids, such as CMTFNet [49], UNetFormer [28], and DC-Swin [27]; and (3) CNN–Mamba hybrids, including PyramidMamba [50], RS3Mamba [38], AfaMamba [45], and UMFormer [51]. To ensure a rigorous evaluation, all models were trained and tested under identical experimental protocols. We employ four standard metrics: per-class F1 score, mean F1 (mF1), Overall Accuracy (OA), and mean Intersection over Union (mIoU). These metrics collectively assess pixel-wise classification precision, global correctness, and region-level segmentation overlap.

**Performance Comparison on the Vaihingen Dataset:** Table 1 presents the quantitative results for the Vaihingen test set. Notably, the proposed MAFMamba sets a new benchmark, achieving state-of-the-art performance across all three comprehensive metrics: mF1 (91.79%), OA (93.66%), and mIoU (85.09%). Compared to the second-best method, AfaMamba [45], our model demonstrates improvements of 0.46%, 0.32%, and 0.73%, respectively. At the category level, MAFMamba secures the highest F1 scores in three classes: Impervious Surface (97.09%), Building (96.30%), and Car (90.40%). Most significantly, it surpasses AfaMamba [45] by 0.96% in the “Car” category, a small target class typically susceptible to scale variations and shadow occlusions. When compared to classical CNN methods—such as ESDINet [47], which achieves an mF1 of only 89.99%—MAFMamba significantly enhances long-range dependency modeling through the integration of the Adaptive Multi-scale Mamba Block (AMMB) in the encoder and global–local interaction in the decoder. Furthermore, relative to CNN–Transformer hybrids like DC-Swin [28], our model achieves superior accuracy with reduced memory consumption. Compared to other Mamba-based baselines, the strategic incorporation of the MSCAF and ASF modules effectively bridges the semantic gap between multi-scale features. This robustness is particularly evident in the model’s ability to distinguish low vegetation from trees and to precisely delineate building boundaries.

**Figure 6:**
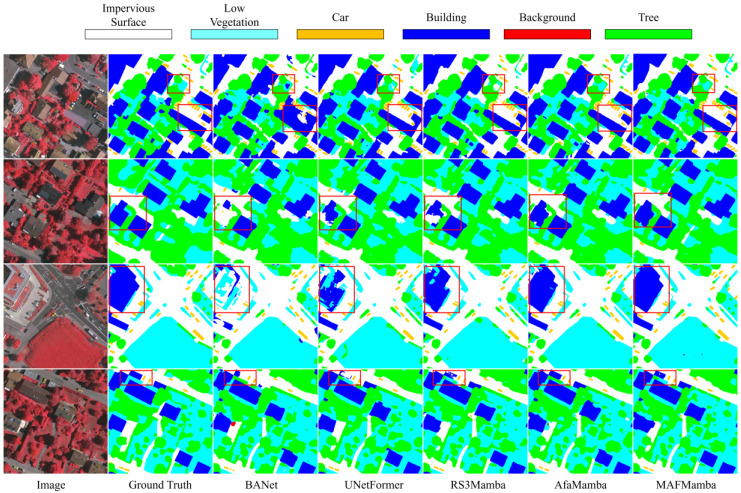
Qualitative comparison of our proposed MAFMamba model against other methods on the Vaihingen dataset (representative regions highlighted with red rectangles).

Qualitative comparisons are illustrated in Figure 6. To ensure visual clarity, we selected representative state-of-the-art methods from different paradigms. As highlighted by the red rectangles, in scenarios characterized by complex shadow occlusions and dense building structures, BANet [48] and UNetFormer [28] exhibit noticeable boundary artifacts and fail to detect small vehicles. While RS3Mamba [38] improves overall coherence, it still misclassifies isolated vehicles and specific low vegetation regions. Similarly, AfaMamba [45] shows confusion between low vegetation and trees in certain areas. In contrast, the segmentation maps produced by MAFMamba display the smoothest boundaries, the most comprehensive detail preservation, the highest detection accuracy for small vehicles, and the clearest distinction between low vegetation and trees. This vividly demonstrates the model’s superior capability in balancing global–local features and aligning multi-scale information.

**Performance Comparison on the Potsdam Dataset:** Table 2 details the quantitative results for the Potsdam test set. Consistently, MAFMamba achieves state-of-the-art performance, recording an mF1 of 92.83%, OA of 91.35%, and mIoU of 86.89%. It outperforms the runner-up, UMFormer [51], by margins of 0.11%, 0.22%, and 0.59% in mF1, OA, and mIoU, respectively, and surpasses AfaMamba [45] by 0.34%, 0.40%, and 0.63%. Regarding per-class performance, MAFMamba obtains the highest F1 scores in three categories: Low Vegetation (88.29%), Tree (90.23%), and Car (96.71%). The substantial lead of 1.20% over UMFormer [51] on the challenging “Tree” class can be attributed to the adaptive cross-scale texture recalibration within the AMMB encoder and the effective global–local feature interplay in the GLMamba decoder.

Compared to traditional CNN methods, MAFMamba markedly reduces intra-class confusion. Against CNN–Transformer hybrids like CMTFNet [49], its linear-complexity state-space modeling ensures higher inference efficiency. Furthermore, relative to other Mamba-based approaches, the MSCAF module enables more precise multi-scale semantic alignment via efficient cross-attention. This results in superior segmentation fidelity in fine-grained regions, such as road-impervious surface boundaries and intricate tree canopy structures.

**Figure 7:**
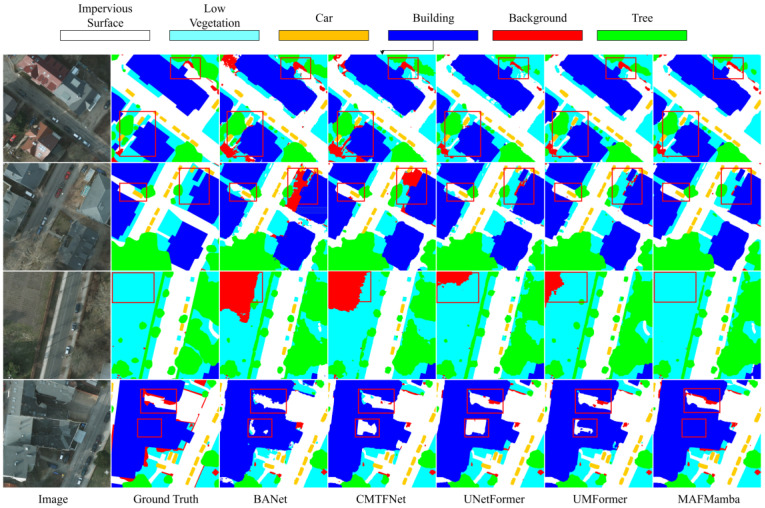
Qualitative comparison of our proposed MAFMamba model against other methods on the Potsdam dataset (representative regions highlighted with red rectangles).

Qualitative results are presented in Figure 7. We again selected representative baselines to verify model robustness. In typical urban scenes featuring interleaved road networks and vegetation, BANet [48] and CMTFNet [49] exhibit frequent misclassifications at road edges and within background clutter. Although UNetFormer [28] successfully outlines large-scale buildings, its reconstruction of internal tree structures remains relatively coarse. UMFormer [51] also retains noise in background areas. Conversely, the prediction map generated by MAFMamba most closely resembles the Ground Truth, featuring sharp road boundaries, complete internal canopy textures, and minimal background noise. This is particularly notable in complex intersections and densely vegetated areas (highlighted by red boxes), demonstrating remarkable detail recovery and discriminative capability. These advantages stem primarily from the multi-scale adaptive fusion module and the hierarchical feature reconstruction strategy in the decoder, which enable a more refined integration of spatial structures and semantic relationships.

**Performance Comparison on the LoveDA Dataset:** To assess the model’s generalizability across varying spatial resolutions and diverse landscape types, we further evaluated MAFMamba on the LoveDA test set. The quantitative results are presented in Table 3. In contrast to the urban-centric ISPRS benchmarks, LoveDA features a lower spatial resolution (0.3 m) and poses unique challenges due to its inclusion of complex rural and agricultural scenes, characterized by large-scale unstructured textures (e.g., farmlands, forests, and water bodies) and sparse building distributions.

As shown in Table 3, MAFMamba achieves top-tier performance with an mIoU of 53.46%, surpassing the runner-up UMFormer [51] and AfaMamba [45] by 0.97% and 1.14%, respectively. Notably, our model demonstrates exceptional robustness in non-urban categories, obtaining the highest scores in Agriculture (64.51%), Water (81.47%), and Forest (46.81%). Specifically, compared to RS3Mamba [38], our method improves accuracy for the Agriculture class by 3.14%. This suggests that the proposed Multi-Scale Adaptive Fusion (MSAF) mechanism effectively aggregates multi-level semantics to resolve the scale variations inherent in vast farmland and natural terrains. Furthermore, in the Building category, which involves sparse and multi-scale rural structures, MAFMamba achieves a leading score of 59.26%, outperforming the CNN-based MANet [19] by 3.06%. These results confirm that MAFMamba possesses strong generalizability, maintaining high segmentation fidelity not only in dense urban areas but also in complex rural environments with varying image resolutions.

**Figure 8:**
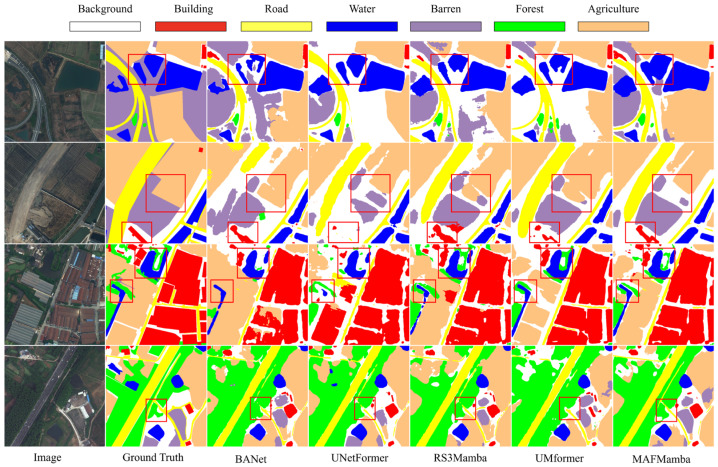
Qualitative comparison of our proposed MAFMamba model against other methods on the LoveDA dataset (representative regions highlighted with red rectangles).

Qualitative comparisons for LoveDA are provided in Figure 8. As observed, BANet [48] and UNetFormer [28] exhibit noticeable misclassifications in complex transition zones; for instance, they fail to accurately delineate the irregular boundaries of water bodies (Row 1) and often confuse barren land with agriculture (Row 2). While Mamba-based methods like RS3Mamba [38] and UMFormer [51] improve global coherence, they still retain “salt-and-pepper” noise in regions with scattered objects, such as the sparse building areas in Row 3. In contrast, MAFMamba produces segmentation maps closest to the Ground Truth, yielding sharp, continuous boundaries for rural water bodies and effectively reconstructing the complete shapes of sparse buildings with minimal background noise. This demonstrates the model’s superior capability in handling the unstructured details of rural imagery, largely attributed to the AMMB module’s ability to strictly balance local fine-grained features with global context.

### 3.4. Ablation Experiments

To systematically validate the efficacy and necessity of each core component within the proposed MAFMamba model, we conducted a comprehensive series of ablation studies on the ISPRS Vaihingen dataset. To ensure a rigorous comparison, all ablation variants were trained and evaluated under identical protocols and parameter settings (e.g., input resolution, optimizer, and data augmentation strategies) as detailed in Section 3.1. As evidenced in Table 4, the complete MAFMamba model—comprising the AMMB, GLMamba, ASF, and MSCAF modules—achieved optimal performance across three key metrics: mF1, Overall Accuracy (OA), and mean Intersection over Union (mIoU). This performance significantly surpasses that of all ablated variants, fully demonstrating the superiority of the holistic architectural design.

The specific contribution of each module is analyzed as follows:

1. **Impact of AMMB:** The exclusion of the Adaptive Multi-scale Mamba Block (w/o AMMB) precipitated decreases of 0.44%, 0.81%, and 0.54% in mF1, OA, and mIoU, respectively. This decline underscores the critical role of the AMMB in modeling multi-scale semantics and long-range dependencies. By degrading the encoder to a standard ResNet-18 architecture stripped of AMMB, the model loses the capability for dynamic multi-scale feature adjustment, significantly weakening the representation of cross-scale objects such as “Tree” and “Low Vegetation”.
2. **Impact of GLMamba:** The omission of the GLMamba module resulted in a 0.38% decrease in mF1 and a 0.47% decrease in mIoU. GLMamba is designed to explicitly model the interaction between global and local contexts at the decoder end. Without this module, the decoder relies solely on conventional upsampling and feature concatenation, struggling to effectively balance high-resolution spatial details with low-resolution deep semantics. Consequently, this led to increased misdetection rates for small objects like cars and heightened edge blurring in building segmentation.
3. **Impact of VSS (w/o AMMB & GLMamba):** The concurrent removal of both AMMB and GLMamba—effectively eliminating all Mamba components to retain only a lightweight ResNet-18 encoder and standard decoder path—resulted in the most severe performance degradation: mF1 dropped by 0.87% and mIoU by 1.20%. This outcome unequivocally demonstrates the pivotal role of the Visual State Space (VSS) model within MAFMamba. Its ability to model long-range dependencies with linear complexity constitutes the foundational basis for the model’s superiority over traditional CNN–Transformer hybrid architectures. Absent the VSSLayer, the network fails to effectively capture the pervasive global context and cross-scale semantic correlations inherent in remote sensing imagery.
4. **Impact of MSCAF:** The absence of the MSCAF module caused mF1 to decrease by 0.27% and mIoU by 0.41%. MSCAF supersedes conventional skip connections, enabling bidirectional information aggregation and semantic alignment of multi-scale features via efficient cross-attention. Its removal reduces the model to simple feature concatenation, creating a significant semantic gap between high-resolution features in shallow encoder layers and deep decoder features. This results in markedly reduced boundary accuracy for the “Impervious Surface” and “Building” categories.
5. **Impact of ASF:** Finally, removing the ASF module yielded a 0.19% decrease in mF1 and a 0.27% decrease in mIoU. ASF assigns adaptive spatial weights to features of differing resolutions during decoding, thereby enhancing fusion stability. Although MSCAF facilitates cross-scale interaction, the lack of pixel-level dynamic weighting in the absence of ASF reduces the consistency of merging high-resolution details with low-resolution semantics, particularly affecting the distinction between “Low Vegetation” and “Tree”.

Our proposed MAFMamba model achieves an optimal balance between global semantics, local details, and computational efficiency through the organic integration of multi-scale adaptive fusion, global–local interactions, and an efficient cross-scale attention mechanism. Consequently, it delivers the best segmentation performance on the Vaihingen dataset.

To further substantiate the rationale behind our architectural choices, we conducted additional comparative experiments focusing on two key aspects: the selection of the attention mechanism within the MSAF module and the choice of the encoder backbone.

We investigated the influence of different attention mechanisms within the MSAF module by comparing the adopted CA with the Squeeze-and-Excitation (SE) Block and Convolutional Block Attention Module (CBAM). Quantitative results presented in Table 5 indicate that the SE-Block, despite being the most lightweight (29.28 M parameters), yields the lowest mIoU of 85.04% due to its inability to capture spatial structural information. Although CBAM improves mIoU to 85.07% by incorporating a spatial attention branch with large 7 ×7 convolutions, it increases parameter complexity to 29.58 M and computational overhead to 56.45 GFLOPs. In contrast, our method utilizing Coordinate Attention achieves the superior segmentation performance of 85.09%. Unlike CBAM, CA captures long-range spatial dependencies along precise positional directions without relying on heavy convolution operations, maintaining a parameter count (29.39 M) and computational cost (56.19 GFLOPs) comparable to the lightweight SE-Block. This confirms that Coordinate Attention offers the optimal trade-off between spatial sensitivity and model complexity for remote sensing tasks.

Furthermore, we evaluated the impact of the encoder backbone by comparing our ResNet-18-based architecture against a deeper ResNet-50 and a Transformer-based Swin-T, as detailed in Table 6. The results underscore a distinct trade-off between accuracy and efficiency. While the Transformer-based Swin-T achieves the highest accuracy (85.19% mIoU) and ResNet-50 follows closely (85.16% mIoU), the performance gains over our method are marginal, at only +0.10% and +0.07%, respectively. However, these marginal improvements come at a substantial computational cost. Swin-T requires the largest number of parameters (71.82 M), and ResNet-50 nearly doubles the FLOPs to 108.55 G compared to our method. Consequently, our MAFMamba with ResNet-18 achieves a highly competitive mIoU of 85.09% while requiring only 29.39 M parameters and significantly lower computational resources. This justifies our selection of ResNet-18 to ensure the linear complexity of the proposed Mamba architecture, demonstrating that simply scaling up the backbone yields diminishing returns in accuracy while severely compromising efficiency.

### 3.5. Complexity Analysis

To validate the efficiency of the proposed framework, we compared MAFMamba against state-of-the-art methods on the Vaihingen dataset in terms of Parameters, GFLOPs, and mIoU. The comparative methods are categorized into three paradigms: CNN-based (C), CNN–Transformer (C–T), and CNN–Mamba (C–M).

As illustrated in Table 7, distinct trade-offs are observed across the different architectures. Among CNN-based methods, traditional models such as BANet [48] serve as the most lightweight baselines (13.06 GFLOPs). However, due to their limited global modeling capabilities, they yield the lowest accuracy (81.35%). Conversely, while MANet [19] increases model size, it achieves only disproportionate performance gains. Regarding hybrid architectures, UNetFormer [28] offers a reasonable balance between speed and accuracy. However, heavy-weight Transformers like DC-Swin [27] achieve higher accuracy (83.22%) only at the cost of significant computational overhead (112.16 GFLOPs) and increased model size. Existing Mamba-based approaches also struggle to achieve an optimal balance between efficiency and performance. For instance, RS3Mamba [38] and PyramidMamba [50] deliver competitive segmentation results but are computationally expensive, requiring over 300 GFLOPs. Notably, although AfaMamba [45] features a low parameter count (13.01 M), its computational complexity remains high (112.16 GFLOPs), while UMFormer [51] falls behind in segmentation accuracy despite its efficiency.

In contrast, MAFMamba secures the highest segmentation performance (85.09% mIoU) while maintaining a highly efficient computational footprint (56.19 GFLOPs). This result confirms that the proposed multi-scale adaptive fusion architecture successfully maximizes performance without incurring high computational costs.

## 4. Conclusions

This paper presents MAFMamba, a Multi-scale Adaptive Fusion visual Mamba network designed for the semantic segmentation of high-resolution remote sensing imagery. It effectively resolves the inherent trade-off between preserving local details and modeling global context found in existing approaches, while simultaneously mitigating the challenges posed by extreme scale variations. MAFMamba employs a hybrid encoder built upon ResNet-18 and VMamba. Central to this architecture is the proposed Adaptive Multi-scale Mamba Block (AMMB), which dynamically aggregates and recalibrates cross-level features via a Multi-Scale Adaptive Fusion (MSAF) mechanism. This operates in conjunction with the VSSLayer to achieve efficient multi-scale semantic extraction and long-range dependency modeling. For the decoder, we designed a Global–Local Feature Enhancement Mamba (GLMamba) and an Adaptive Scale Fusion (ASF) module. Crucially, we introduced the Multi-Scale Cross-Attention Fusion (MSCAF) module to explicitly align fine-grained encoder features with high-level decoder semantics, thereby effectively bridging the semantic gap across scales.

Extensive experiments on the ISPRS Potsdam, Vaihingen, and LoveDA datasets demonstrate that MAFMamba establishes new state-of-the-art performance. It achieves an mIoU of 85.09% on Vaihingen and 86.89% on Potsdam, surpassing leading CNN, Transformer, and other Mamba-based methods. Notably, performance on the LoveDA dataset (53.46% mIoU) confirms the model’s strong generalizability and robustness when processing complex rural agricultural scenes and imagery with varying spatial resolutions. Furthermore, complexity analysis confirms that MAFMamba achieves an optimal trade-off between accuracy and efficiency, delivering superior segmentation performance while maintaining a significantly lower computational footprint (in terms of Parameters and GFLOPs) compared to heavy Transformer-based architectures.

Notwithstanding these advancements, MAFMamba exhibits limitations in handling densely packed small objects and areas under heavy shadow occlusion, where boundary ambiguity and insufficient contextual reasoning from occluded regions remain challenging. Future research directions include refining boundary-aware learning mechanisms, enhancing feature representation for occlusion scenarios, and employing more rigorous validation protocols to further improve generalization. Overall, this work presents a novel and effective paradigm, offering a promising technical pathway for advancing efficient Mamba-based architectures in remote sensing semantic segmentation.

## Figures and Tables

**Table 1:** Quantitative comparison of different methods on the Vaihingen dataset (best results in bold). All metrics for the categories are F1 scores.

Mothod	Imp. Surf.	Building	Low Veg.	Tree	Car	mF1 (%)	OA (%)	mIoU (%)
BANet [48]	92.23	95.23	83.75	89.92	86.76	89.58	90.48	81.35
ESDINet [47]	92.74	95.54	84.46	90.03	87.16	89.99	90.88	82.03
MANet [19]	93.01	95.43	84.62	89.96	88.93	90.39	90.93	82.71
UNetFormer [28]	92.72	95.34	84.91	**90.68**	88.53	90.43	92.21	82.89
DC-Swin [27]	93.60	96.18	**85.75**	90.36	87.64	90.71	91.63	83.22
PyramidMamba [50]	96.99	96.09	84.80	89.95	88.64	91.30	93.50	84.32
RS3Mamba [38]	96.70	95.50	84.40	90.00	86.90	90.73	92.27	83.30
AfaMamba [45]	96.81	95.90	84.51	89.98	89.44	91.33	93.34	84.36
UMFormer [51]	96.64	95.47	84.48	89.85	88.81	91.05	93.11	83.89
MAFMamba (ours)	**97.09**	**96.30**	84.82	90.37	**90.40**	**91.79**	**93.66**	**85.09**

**Table 2:** Quantitative comparison of different methods on the Potsdam dataset (best results in bold). All metrics for the categories are F1 scores.

Method	Imp. Surf.	Building	Low Veg.	Tree	Car	mF1 (%)	OA (%)	mIoU (%)
BANet [48]	91.42	95.65	85.67	86.88	91.40	90.20	89.14	82.35
ESDINet [47]	92.68	96.28	87.29	88.10	95.42	91.96	90.53	85.32
MANet [19]	92.41	96.13	87.08	88.14	95.32	91.81	90.30	85.06
UnetFormer [28]	93.45	94.69	86.31	87.72	96.28	91.69	89.94	84.90
CMTFNet [49]	92.12	96.41	86.43	87.26	92.41	90.93	89.89	83.57
PyramidMamba [50]	92.18	95.91	87.33	88.87	95.89	92.94	90.50	85.45
RS3Mamba [38]	**94.21**	94.72	85.95	88.11	96.00	91.80	90.07	85.09
AfaMamba [45]	93.04	96.35	87.67	88.74	96.67	92.49	90.95	86.26
UMFormer [51]	93.37	**97.09**	86.70	89.03	96.34	92.72	91.13	86.30
MAFMamba (ours)	93.12	96.80	**88.29**	**90.23**	**96.71**	**92.83**	**91.35**	**86.89**

**Table 3:** Quantitative comparison of different methods on the LoveDA dataset (best results in bold). All metrics for the categories are F1 scores.

Method	Background	Building	Road	Water	Barren	Forest	Agriculture	mIoU (%)
BANet [48]	41.92	54.13	52.39	76.18	11.74	45.07	51.71	47.59
MANet [19]	44.38	56.20	56.03	78.41	17.06	45.81	58.17	50.87
UNetFormer [28]	44.60	55.59	52.85	77.80	16.19	43.15	58.36	49.64
CMTFNet [49]	40.71	49.18	50.63	76.95	14.32	41.84	54.19	47.59
PyramidMamba [50]	45.60	56.37	54.26	80.33	15.77	46.06	61.73	51.45
RS3Mamba [38]	41.60	58.23	54.03	77.34	17.97	43.81	61.37	50.62
AfaMamba [45]	45.15	58.64	54.78	80.12	17.96	44.87	62.86	52.32
UMFormer [51]	45.48	58.73	56.51	80.41	**18.89**	45.47	62.93	52.49
MAFMamba (Ours)	**46.88**	**59.26**	**56.84**	**81.47**	18.25	**46.81**	**64.51**	**53.46**

**Table 4:** Ablation study of different components on the Vaihingen dataset (best results in bold; ✓: Selected; ✗: Not selected).

Method	MSCAF	GLMamba	ASF	AMMB	mF1 (%)	OA (%)	mIOU (%)
MAFMamba	✓	✓	✓	✓	**91.79**	**93.66**	**85.09**
w/o AMMB	✓	✓	✓	✗	91.35	92.85	84.55
w/o GLMamba	✓	✗	✓	✓	91.41	92.73	84.62
w/o AMMB & GLMamba	✓	✗	✓	✗	90.92	92.16	83.89
w/o MSCAF	✗	✓	✓	✓	91.52	93.35	84.68
w/o ASF	✓	✓	✗	✓	91.60	93.42	84.82

**Table 5:** Ablation study of attention mechanisms on the Vaihingen dataset (best results in bold).

Mothod	Attention Type	Params (M)	GFLOPs	mF1 (%)	OA (%)	mIOU (%)
MAFMamba w/SE	Channel	**29.28**	**56.12**	91.75	93.63	85.04
MAFMamba w/CBAM	Channel + Spatial	29.58	56.45	91.77	93.65	85.07
MAFMamba (Ours)	Coordinate	29.39	56.19	**91.79**	**93.66**	**85.09**

**Table 6:** Comparison of backbone networks on the Vaihingen dataset (best results in bold).

Backbone	Type	Params (M)	GFLOPs	mF1 (%)	OA (%)	mIOU (%)
Swin-T	Transformer	71.82	97.45	**91.85**	**93.73**	**85.19**
ResNet-50	CNN	68.55	108.55	91.83	93.71	85.16
ResNet-18 (Ours)	CNN	**29.39**	**56.19**	91.79	93.66	85.09

**Table 7:** Efficiency analysis on the Vaihingen dataset (best results in bold).

Method	Type	Params (M)	GFLOPs	mIOU (%)
BANet [48]	C	12.73	**13.06**	81.35
MANet [19]	C	35.86	77.62	82.71
UNetFormer [28]	C-T	**11.68**	46.97	82.89
DC-Swin [27]	C-T	45.60	112.16	83.22
PyramidMamba [50]	C-M	115.12	398.58	84.32
RS3Mamba [38]	C-M	51.00	300.76	83.30
AfaMamba [45]	C-M	13.01	112.16	84.36
UMFormer [51]	C-M	12.49	47.76	83.89
MAFMamba (Ours)	C-M	29.39	56.19	**85.09**

## Data Availability

The data presented in this study are available on request from the corresponding author.

## References

[1] Yang Y., Sun X., Dong J., Lam K.-M., Zhu X.X. (2024). Attention-ConvNet Network for Ocean-Front Prediction via Remote Sensing SST Images. IEEE Trans. Geosci. Remote Sens..

[2] Xiang S., Xie Q., Wang M. (2022). Semantic Segmentation for Remote Sensing Images Based on Adaptive Feature Selection Network. IEEE Geosci. Remote Sens. Lett..

[3] Xu C., Du X., Fan X., Yan Z., Kang X., Zhu J., Hu Z. (2022). A Modular Remote Sensing Big Data Framework. IEEE Trans. Geosci. Remote Sens..

[4] Sun X., Zhang M., Dong J., Lguensat R., Yang Y., Lu X. (2021). A Deep Framework for Eddy Detection and Tracking from Satellite Sea Surface Height Data. IEEE Trans. Geosci. Remote Sens..

[5] He X., Zhou Y., Zhao J., Zhang D., Yao R., Xue Y. (2022). Swin Transformer Embedding UNet for Remote Sensing Image Semantic Segmentation. IEEE Trans. Geosci. Remote Sens..

[6] Xin Y., Fan Z., Qi X., Zhang Y., Li X. (2024). Confidence-Weighted Dual-Teacher Networks with Biased Contrastive Learning for Semi-Supervised Semantic Segmentation in Remote Sensing Images. IEEE Trans. Geosci. Remote Sens..

[7] Peláez-Vegas A., Mesejo P., Luengo J. (2023). A Survey on Semi-Supervised Semantic Segmentation. arXiv.

[8] Zheng Y., He L., Wu X., Pan C. (2023). Self-Training and Multi-Level Adversarial Network for Domain Adaptive Remote Sensing Image Segmentation. Neural Process. Lett..

[9] Liu J., Hua W., Zhang W., Liu F., Xiao L. (2024). Stair Fusion Network with Context-Refined Attention for Remote Sensing Image Semantic Segmentation. IEEE Trans. Geosci. Remote Sens..

[10] Cai X., Lai Q., Wang Y., Wang W., Sun Z., Yao Y. Poly Kernel Inception Network for Remote Sensing Detection. Proceedings of the IEEE/CVF Conference on Computer Vision and Pattern Recognition (CVPR).

[11] Long J., Shelhamer E., Darrell T. Fully Convolutional Networks for Semantic Segmentation. Proceedings of the IEEE Conference on Computer Vision and Pattern Recognition (CVPR).

[12] Ronneberger O., Fischer P., Brox T., Navab N., Hornegger J., Wells W.M., Frangi A.F. (2015). U-Net: Convolutional Networks for Biomedical Image Segmentation. Medical Image Computing and Computer-Assisted Intervention—MICCAI 2015.

[13] Li R., Zheng S., Duan C., Su J., Zhang C. (2022). Multistage Attention ResU-Net for Semantic Segmentation of Fine-Resolution Remote Sensing Images. IEEE Geosci. Remote Sens. Lett..

[14] Wu X., Hong D., Chanussot J. (2022). Convolutional Neural Networks for Multimodal Remote Sensing Data Classification. IEEE Trans. Geosci. Remote Sens..

[15] Zhang X., Zhang B., Yu W., Kang X. (2023). Federated Deep Learning with Prototype Matching for Object Extraction from Very-High-Resolution Remote Sensing Images. IEEE Trans. Geosci. Remote Sens..

[16] Ma X., Zhang X., Pun M.-O., Liu M. (2024). A Multilevel Multimodal Fusion Transformer for Remote Sensing Semantic Segmentation. IEEE Trans. Geosci. Remote Sens..

[17] Chen L.-C., Zhu Y., Papandreou G., Schroff F., Adam H., Ferrari V., Hebert M., Sminchisescu C., Weiss Y. (2018). Encoder-Decoder with Atrous Separable Convolution for Semantic Image Segmentation. Computer Vision–ECCV 2018.

[18] Li R., Zheng S., Zhang C., Duan C., Wang L., Atkinson P.M. (2021). ABCNet: Attentive Bilateral Contextual Network for Efficient Semantic Segmentation of Fine-Resolution Remotely Sensed Imagery. ISPRS J. Photogramm. Remote Sens..

[19] Li R., Zheng S., Zhang C., Duan C., Su J., Wang L., Atkinson P.M. (2022). Multiattention Network for Semantic Segmentation of Fine-Resolution Remote Sensing Images. IEEE Trans. Geosci. Remote Sens..

[20] Zunair H., Hamza A.B. Masked Supervised Learning for Semantic Segmentation. Proceedings of the 33rd British Machine Vision Conference (BMVC).

[21] Yang G., Lei J., Tian H., Feng Z., Liang R. (2024). Asymptotic Feature Pyramid Network for Labeling Pixels and Regions. IEEE Trans. Circuits Syst. Video Technol..

[22] Yang C., Chen M., Xiong Z., Yuan Y., Wang Q. (2022). CM-Net: Concentric Mask Based Arbitrary-Shaped Text Detection. IEEE Trans. Image Process..

[23] Yang C., Chen M., Yuan Y., Wang Q. (2024). Zoom Text Detector. IEEE Trans. Neural Netw. Learn. Syst..

[24] Dosovitskiy A., Beyer L., Kolesnikov A., Weissenborn D., Zhai X., Unterthiner T., Dehghani M., Minderer M., Heigold G., Gelly S. (2020). An Image Is Worth 16x16 Words: Transformers for Image Recognition at Scale. arXiv.

[25] Strudel R., Garcia R., Laptev I., Schmid C. Segmenter: Transformer for Semantic Segmentation. Proceedings of the IEEE/CVF International Conference on Computer Vision (ICCV).

[26] Xie E., Wang W., Yu Z., Anandkumar A., Alvarez J.M., Luo P. (2021). SegFormer: Simple and Efficient Design for Semantic Segmentation with Transformers. arXiv.

[27] Wang L., Li R., Duan C., Zhang C., Meng X., Fang S. (2022). A Novel Transformer Based Semantic Segmentation Scheme for Fine-Resolution Remote Sensing Images. IEEE Geosci. Remote Sens. Lett..

[28] Wang L., Li R., Zhang C., Fang S., Duan C., Meng X., Atkinson P.M. (2022). UNetFormer: A UNet-like Transformer for Efficient Semantic Segmentation of Remote Sensing Urban Scene Imagery. Heliyon.

[29] Liu Z., Lin Y., Cao Y., Hu H., Wei Y., Zhang Z., Lin S., Guo B. (2021). Swin Transformer: Hierarchical Vision Transformer Using Shifted Windows. Proceedings of the IEEE/CVF International Conference on Computer Vision (ICCV).

[30] Kitaev N., Kaiser Ł., Levskaya A. (2020). Reformer: The Efficient Transformer. arXiv.

[31] Beltagy I., Peters M.E., Cohan A. (2020). Longformer: The Long-Document Transformer. arXiv.

[32] Gu A., Dao T. (2023). Mamba: Linear-Time Sequence Modeling with Selective State Spaces. arXiv.

[33] Zhu L., Liao B., Zhang Q., Wang X., Liu W., Wang X. (2024). Vision Mamba: Efficient Visual Representation Learning with Bidirectional State Space Model. arXiv.

[34] Liu Y., Tian Y., Zhao Y., Yu H., Xie L., Wang Y., Ye Q., Jiao J., Liu Y. (2024). VMamba: Visual State Space Model. arXiv.

[35] Yang C., Chen Z., Espinosa M., Ericsson L., Wang Z., Liu J., Crowley E.J. (2024). PlainMamba: Improving Non-Hierarchical Mamba in Visual Recognition. arXiv.

[36] Hatamizadeh A., Kautz J. (2024). MambaVision: A Hybrid Mamba-Transformer Vision Backbone. arXiv.

[37] Zhu Q., Cai Y., Fang Y., Yang Y., Chen C., Fan L., Nguyen A. (2024). Samba: Semantic Segmentation of Remotely Sensed Images with State Space Model. Heliyon.

[38] Ma X., Zhang X., Pun M.-O. (2024). RS3 Mamba: Visual State Space Model for Remote Sensing Image Semantic Segmentation. IEEE Geosci. Remote Sens. Lett..

[39] Liu M., Dan J., Lu Z., Yu Y., Li Y., Li X. (2024). CM-UNet: Hybrid CNN-Mamba UNet for Remote Sensing Image Semantic Segmentation. arXiv.

[40] He X., Cao K., Zhang J., Yan K., Wang Y., Li R., Xie C., Hong D., Zhou M. (2025). Pan-Mamba: Effective Pan-Sharpening with State Space Model. Inf. Fusion.

[41] Chen H., Song J., Han C., Xia J., Yokoya N. (2024). ChangeMamba: Remote Sensing Change Detection with Spatiotemporal State Space Model. IEEE Trans. Geosci. Remote Sens..

[42] Liu L., Zhang M., Yin J., Liu T., Ji W., Piao Y., Lu H. (2025). DefMamba: Deformable Visual State Space Model. arXiv.

[43] Wang G., Zhang X., Zhang Y., Peng Z., Zhang T., Tang X., Jiao L. (2025). ACMamba: Fast Unsupervised Anomaly Detection via An Asymmetrical Consensus State Space Model. arXiv.

[44] Liu S., Huang D., Wang Y. (2019). Learning Spatial Fusion for Single-Shot Object Detection. arXiv.

[45] Chen H., Luo H., Wang C. (2025). AfaMamba: Adaptive Feature Aggregation with Visual State Space Model for Remote Sensing Images Semantic Segmentation. IEEE J. Sel. Top. Appl. Earth Obs. Remote Sens..

[46] Zeng Q., Zhou J., Tao J., Chen L., Niu X., Zhang Y. (2024). Multiscale Global Context Network for Semantic Segmentation of High-Resolution Remote Sensing Images. IEEE Trans. Geosci. Remote Sens..

[47] Zhang X., Weng Z., Zhu P., Han X., Zhu J., Jiao L. (2024). ESDINet: Efficient Shallow-Deep Interaction Network for Semantic Segmentation of High-Resolution Aerial Images. IEEE Trans. Geosci. Remote Sens..

[48] Wang L., Li R., Wang D., Duan C., Wang T., Meng X. (2021). Transformer Meets Convolution: A Bilateral Awareness Network for Semantic Segmentation of Very Fine Resolution Urban Scene Images. Remote Sens..

[49] Wu H., Huang P., Zhang M., Tang W., Yu X. (2023). CMTFNet: CNN and Multiscale Transformer Fusion Network for Remote-Sensing Image Semantic Segmentation. IEEE Trans. Geosci. Remote Sens..

[50] Wang L., Li D., Dong S., Meng X., Zhang X., Hong D. (2025). PyramidMamba: Rethinking Pyramid Feature Fusion with Selective Space State Model for Semantic Segmentation of Remote Sensing Imagery. Int. J. Appl. Earth Obs. Geoinf..

[51] Li L., Yi J., Fan H., Lin H. (2025). A Lightweight Semantic Segmentation Network Based on Self-Attention Mechanism and State Space Model for Efficient Urban Scene Segmentation. IEEE Trans. Geosci. Remote Sens..

